# UK podiatrists’ experiences of podiatry services for people living with arthritis: a qualitative investigation

**DOI:** 10.1186/s13047-018-0262-5

**Published:** 2018-06-05

**Authors:** Louise McCulloch, Alan Borthwick, Anthony Redmond, Katherine Edwards, Rafael Pinedo-Villanueva, Daniel Prieto-Alhambra, Andrew Judge, Nigel K. Arden, Catherine J. Bowen

**Affiliations:** 1 Faculty of Health Sciences, University of Southampton, Highfield Campus Building 45, University Road, Southampton, Hampshire SO17 1BJ UK; 2 Faculty of Medicine & Health, Leeds Institute of Rheumatic & Musculoskeletal Medicine, Leeds, UK; 3 Nuffield Department of Orthopaedics, Rheumatology & Musculoskeletal Sciences, University of Oxford, Oxford, UK

**Keywords:** Arthritis, Podiatry, Footcare, Service provision

## Abstract

**Background:**

Provision of podiatry services, like other therapies in the UK, is an area that lacks guidance by the National Institute for Health and Care Excellence. Many individuals living with arthritis in the UK are not eligible to access NHS podiatry services. The primary aim of this investigation was to understand the views of podiatry clinicians on their experiences of referral, access, provision and treatment for foot problems for patients who have arthritis.

**Methods:**

Focus groups were undertaken to explore, in-depth, individual views of podiatrists working in the UK to gain feedback on experiences of barriers and facilitators to referral, access, provision and treatment for foot problems for individuals living with arthritis. A purposive sampling strategy was adopted and two, semi-structured, focus group interviews conducted, involving 12 podiatrists from both NHS and independent sectors. To account for geographical variations one focus group took place in each of 2 predetermined ‘zones’ of the UK; Yorkshire and Hampshire. Thematic analysis was employed to identify key meanings and report patterns within the data.

**Results:**

The key themes derived from the podiatry clinician focus groups suggest a variety of factors influencing demand for, and burden of, foot pain within the UK. Participants expressed frustration on having a service that accepts and treats patients according to their condition, rather than their complaint. Additionally, concern was conveyed over variations in the understanding of stakeholders’ views of what podiatry is and what podiatrists aim to achieve for patients.

**Conclusion:**

Podiatrists interviewed believed that many individuals living with arthritis in the UK are not eligible to access NHS podiatry services and that this may be, in part, due to confusion over what is known about podiatry and access criteria. Essentially, podiatrists interviewed called for a timely renaissance of current systems, to newer models of care that meet the foot care needs of individual patients’ circumstances and incorporate national multi-disciplinary guidance. Through this project, we have formulated key recommendations that are directed towards improving what other stakeholders (including GPs, commissioners and users of podiatry services) know about the effectiveness of podiatry and also to futureproof the profession of podiatry.

## Background

Provision of podiatry services, like other health therapies in the UK, is an area that lacks guidance by the UK National Institute for Health and Care Excellence. There is a perceived lack of understanding of how to access treatment for foot problems, and limited understanding of what podiatry services can offer, by both patients and non-podiatric clinicians [1–6]. Many podiatry departments have recently felt themselves unsettled by job cuts and recent changes in how services are commissioned has shifted focus towards management of acute wounds, specifically for the management and prevention of limb loss associated with diabetes [7, 8] The consequent impact of podiatry services reconfiguration of skill mix and services, away from management of foot pain associated with other chronic conditions such as rheumatoid arthritis and osteoarthritis, is not known. General Practitioners (GPs) are consulted by 15% of the reported 20 million people in the United Kingdom (UK) with symptoms of rheumatic disease each year, forming up to 25% of a GPs workload [9]; with nearly 30% of the older population in chronic pain due to ‘arthritis’ [10] or a lessened quality of life [11], rheumatological disability is predicted to be a major public health concern in the coming years [12]. This loss of podiatry services from the UK NHS potentially puts the most frail and vulnerable people at risk of mobility loss [13, 14]. The primary aim of this investigation was to understand the views of podiatrists on their experiences of referral, access, provision and treatment for foot problems for patients who have arthritis. Arthritis was selected as the long-term condition to scrutinise due to feedback from our patient and public involvement (PPI) consultations as the one that caused the most confusion over access to foot-care.

## Methods

A qualitative research study design was employed to enable a deep exploration of podiatrists’ views, to gain feedback on experiences of barriers and facilitators to referral, access, provision and treatment for foot problems for individuals living with arthritis. Focus groups were chosen as the most appropriate approach to capture a large amount of information in a relatively short period of time [15] and allowed us to not only to identify the issues that the podiatrists’ raised, but also allowed for the observation of how podiatrists discussed the issues in a ‘natural’ social setting. The methods adopted reflected existing standards for robustness in qualitative research, deploying triangulation of data, respondent validation and data saturation, which guided the final sample size [16–18].

### Participants

Participants were recruited through their membership of the College of Podiatry UK. Potential participants were emailed a brief overview of the study through the Colleges’ newswire. Those interested in joining the study were emailed an information sheet, along with the contact details of the primary investigator (LMc). Interested podiatrists then contacted the primary investigator (LMc) for additional information, to have any further questions answered and be screened against the project’s criteria.

A purposive sampling strategy was undertaken, consistent with the qualitative study design adopted. Participants were selected according to time since qualification, employer (NHS, independent or academia) and experience of managing foot health for individual’s living with arthritis, to ensure the study would capture insightful and meaningful data from a diversity of experience, employments and perspectives. To enable a ‘snapshot’ of 2 representative areas of the UK, 2 zones were established; Yorkshire (North England) and Hampshire (South England) and a focus group interview was held in each of the zones.

### Procedure

Each focus group was conducted by the main researcher (LMc) supported by a second investigator (KE in Yorkshire; AB in Hampshire) as note-taker to aid with reflection, transcription and subsequent coding. General topics for discussion were identified with pre-determined ‘topic guide’ questions written prior to the focus groups. The topic guide was informed by, and constructed from, the findings from analysis of a systematic review of the literature relative to evidence for podiatry and foot care conducted by the team [19].

Digital audio-recordings were transcribed, anonymised and imported into a data analysis package (N-Vivo 11). Using this and manual methods, codes were generated by noting recurring comments and used to categorise responses by the researcher (LMc). The codes were refined, compared and grouped into similar features which served as potential themes. Thematic analysis was identified as a suitable method to search for patterns related to podiatrists’ views on podiatry services for individual’s living with arthritis [16, 18]. Emerging themes were discussed by the wider research team (LMc, AB, CB) for verification, identification of any additional areas of interest and consensus via discussion of patterns across the data. Potential themes were repeatedly discussed by the research team to identify any alternative interpretations. The process of verifying themes as a team provided a more rigorous approach, different perspectives and agreement on final themes.

## Results

The study recruited 12 participants in total, six to each focus group. Of the clinical podiatrists interviewed, three were solely NHS employed, three were solely in private practice, six worked in a variety of settings (including three working part-time in academia). NHS bandings (where applicable) ranged from 5 to 8. Fifteen codes were initially identified from the 2 focus group interviews. Key themes were constructed via abductive analysis, and are presented in Table 1 with the subthemes and one exemplar quote. An abridged summary, with excerpts of data drawn from the transcripts, is presented below. Quotes are allocated alphabetical codes where required, for differentiation.

**Table 1:** Key themes emergent from focus group interviews

Themes with key quote	Subthemes
Theme 1: Evolving Professional Culture *“Historically the commissioner’s never quite got around to finishing off writing the specification”*	AHPs understanding of Podiatry Commissioning Patients understanding of Podiatry
Theme 2: ‘Condition vs Complaint’ *“Is it about the patient who’s got diabetes or is it that they’ve got diabetic lower limb complications? Because the two are quite different.”*	Inequalities and eligibility Private sector versus NHS The current bandwagon Importance of Podiatry in Arthritis
Theme 3: Transforming and Sustaining Podiatry *“We really need to go to the top and make podiatry the same as dental care, the same as eye care, the same as hearing, audiology, you know, we’re just off the radar.”*	Equipping Podiatrists Building Podiatry Proposals for future shape of Podiatry

### Theme 1: Evolving professional culture

This theme presents clinical podiatrist’s perceptions on how podiatry has become shaped historically. Whilst current podiatry services are well received and valued, participants vocalised a perception that procurement of services can be based on sketchy knowledge and absent evidence:

*“I know with my locality, the proposal that was sent by the CCG, it was decided that actually they lacked the understanding about podiatry. And so it was, we would buy our local level and then send back to them, because otherwise they didn’t quite comprehend what we did. And so we were able to divide it into our separate areas, like nail surgery, routine care, diabetes and then send it back to them so that they had more of an understanding of what we actually did. So that is a problem.” [Podiatrist: LB1]*

This is confounded by an inherent frustration at a continuing dearth of understanding, of the scope, depth and value of podiatric practice, by non-podiatrists including patients:

*“[HB1]: I think it’s still fairly common, from feedback from staff for …the start of the consultation with a new patient is actually getting them to understand why you want to know this.” You know, “what’s the medication got to do with you” et cetera. And that can actually take up some of the initial time that actually when explaining about why it’s important and actually you know “the feet are actually attached to the rest of your body”, that type of conversation.*

*[HG2]: That’s the “what’s that got to do with my feet?”*

*[HB1]: “Yes… a big sum of that time is actually about just starting to drill down and set the scene with the patient about what we’re trying to achieve. And then on to what they want to achieve. With a bit more understanding why we’re taking medical history and how it’s relevant to what’s happening in their feet.”*

And recent changes and streamlining of NHS management structures was discussed:

*“We’ve lost a lot of that middle-management podiatry managers, we haven’t got anybody really fighting for our service at the moment. And just replying to the comment about NHS practitioners, I’ve never, in the whole time that I’ve practiced podiatry, ever seen such disillusionment. I think that everybody’s burnt out at work, I think that they’re being managed by people that don’t actually understand what’s happening.” [LG1]*

### Theme 2: ‘Condition vs Complaint’

This theme presents podiatrists’ unease on how podiatry varies within current healthcare systems, with inconsistent approaches causing inequality, discrimination and discrepancy. An over reliance on tick boxes, and apparent detriment of clinical autonomy, means that people who are not currently ‘at risk’ (but could potentially be in the future) are able to access podiatry services, however those who have high podiatric risk, but by tick box standards are not classified as such, are thus ‘ineligible’.

*“I’m really reluctant to do that because a) you’ve got people with multi-pathologies and b) is it about the patient who’s got diabetes or is it that they’ve got diabetic lower limb complications? Because the two are quite different. And you know again it’s about back to ‘we shouldn’t just be providing services to people with diabetes, it’s about services for people with lower limb complications’… back to what I said earlier about some services that have got severe restrictions, you know you could be a 27 year old with diabetes playing rugby but you could technically get service because you’ve got diabetes as opposed to actually having a need.” [HB1]*

The introduction of access criteria in practice is reported to have created a culture of exclusion to many vulnerable people, with podiatric clinicians alluding to cultures of ‘condition over complaint’ and ‘postcode lottery’. Podiatrists refer to an unmet need of foot-care and the consequential risk of foot-health deterioration. Confusion was felt to exist, around non-podiatrists’ understanding what a status of high or low risk means. And with non-standardised criteria in use (‘pain’, ‘diabetes’ or specific long-term health conditions) eclectic podiatric services are being provided across the country.

*“They tend to put the commissioned service versus service level agreements. So, a lot of diabetes services are commissioned. So they ‘have’ to provide that service, so even though we, in the acute trust run, you know, quite a full rheumatology foot service, as soon as we’re a man down in diabetes, people get pulled from arthritis clinics, from rheumatology, to cover diabetes. And that is just based on, purely commissioned services versus service level agreements. We have a service level agreement to provide treatment for Rheumatology patients…” [HG1]*

### Theme 3: Transforming and sustaining podiatry

This theme captures practising clinicians’ views for the future shape of podiatry in modern healthcare:

*“It* {resolving current constraints to podiatric access} *is multifaceted; it is conversations with the commissioners about getting specification rights in the first place. It is discussions internally within health trusts around priorities and in some ways protecting what we’ve got …So the only way we can get around that* {current constraints to podiatric access}*, as I can see, is raising the profile of the profession. Raising the knowledge and raising the value of what we do and the cost efficiency of what we do.” [HB1]*

Podiatrists want to see inequalities in service provision eliminated, offering a shift in the priorities of podiatric services to incorporate more long term conditions:

*“Podiatry provides an opportunity to pick up long term conditions in the early stages, so we know that mechanically, arthritis in the foot is the second most common site for presentation so podiatrists can be a guard responsible for aiding, for new diagnosis for a patient and show them …you know some red flags for podiatry to go in, because they’re likely to be the people who see the patients when they present with those conditions…“and I’ve got sore feet” you know… “and actually got sore hands too”. But then it’s about getting the training of podiatrists as well, you know, they not just looking at the feet. If there are some red flags that come up like they do with diabetes…what do they do in class about diagnosing arthritis? We all should be...we shouldn’t just be doing the squeeze test to feet, we should be doing the squeeze test on the hands.” [HG1]*

If eligibility systems are used, they should be set nationally, evidenced, agreed across professional groups and used consistently, whilst embracing expert clinical discretion.

*“We need care pathways … You know, I think we need to subdivide all the things that podiatry offers and have a tick box assessment sheet that we can actually offer to somebody that’s diagnosed with arthritis and make sure that there is an effective referral system…for that patient to know what care is available and what they can expect if they're presenting with certain conditions.” [LG1]*

Clinicians suggested a preference for access to podiatry services being more person-centred, tailored to individuals:

*“I think there certainly needs to be evidence to show that there should be a pathway whereby all these patients get some sort of similar assessment to identify what their initial needs are. And maybe on an annual basis or even a three-yearly basis or something, just to ensure that then things can be identified early on to start actually taking place, whether it be footwear, whether it be on education or whether they need to change medication or whatever else... It has to be put up the agenda.” [LG1]*

They propose a more multidisciplinary, coordinated approach to patient services, specifically requesting that arthritis and other long term health conditions have models of access and guidelines comparable to those for diabetes:

*“Pathways. Referral pathways, just like diabetes ...a bit more streamlined and a bit more easy to access.” [HG3]*

*“Make it more equal over the UK rather than just dependant on personality, and really that sums up what people said – going through more pathways, focussing on multidisciplinary teams.” [HG4]*

*“If we had a more multidisciplinary coordinated approach it would be better for the patient. I know that we have access to all these other professions but sometimes those communications, are blocked, not blocked but they are strained because you’ve got to write a letter, and that’s got to go off, then someone’s got to sign it and… whereas if you had a better, multidisciplinary approach like we do in terms of diabetes then those patients would go through proper routes a lot quicker” [HG1]*

They reported a need to work with other professionals to drive changes for more long term conditions:

*“And it's not just our profession that’ll link with them [public health], for the benefit of our patients because, you know, podiatry is one of them, you could have ENT in there, you could have physio in there, you’ve got other disciplines in there. They can all push this agenda forward and start saying, yes, here’s another one, it's a long-term condition that we need to be doing more for.” [LG1]*

Clinicians believed access to podiatry services should be transformed to fit modern healthcare needs, to meet the needs of patients within an evolving healthcare system, which incorporates building in onward referral to private sector podiatrists, into the NHS service:

*“Can I, can I bring something in there that’s quite important? We actually have a good NHS private practice working relationship in our area, the culture that we historically have had is that when a patient is deemed no longer eligible for treatment, that they’re discharged to the third sector. And you know, if you actually have a dentist, and the NHS can't meet your dental needs, you’re recommended that you can seek dentistry privately, the same with seeing an optician.” [LG1]*

## Discussion

Using focus group methodology and a thematic approach to data analysis, this study has provided unique insights into UK podiatrists’ (based in 2 distinct regions of the UK, Yorkshire and Hampshire) perceptions of barriers and facilitators to referral, access, provision and treatment for foot problems for individuals living with arthritis. Our overarching findings indicate that podiatrists experience frustration about the role and status of podiatric services, the inequalities in service provision (between regions and between individuals) and the loss of clinical autonomy – fuelling an ethos of ‘condition over complaint’. The literature relating to foot healthcare supports the need to transform and shape podiatry by promoting the scope of practice, taking ownership of ‘foot care’ and embedding evidence within national and local guidelines [19–22]. The resultant key themes constructed from our investigation are discussed below:

### Evolving professional culture

Notably, podiatrists expressed key concerns of frustration that, although podiatry has evolved as a profession, there remains a sense of misunderstanding, by non-podiatrists and patients, of the scope of practice and ability of podiatrists in what they do. The revelation that podiatrists believe their scope of practice is limited by the profile and image of the profession is not a new one. Earlier work has identified the hierarchical nature of the health professions [21] and the way in which podiatry has perceived itself as less visible and more misunderstood than other comparable professions [22–24]. In our investigation, advances in scope of practice and a growing presence in multi-disciplinary team (MDT) working were clearly considered important factors in raising the profile of the podiatry profession, however were perceived more evident in specific fields, such as diabetes foot care, and much less clear in primary care which is also evidenced widely in the literature [25].

Key leaders in the profession, locally and nationally, were lauded for the development of services in the past and concern was voiced over the increasing trend for managers responsible for defending, promoting and commissioning podiatry services in today’s healthcare practices, to be non-podiatrists with limited knowledge of the scope of practice of the profession. The vital role of key, charismatic characters in podiatry has been previously reported in the literature as being fundamental in developing and augmenting the profession in specialisms such as diabetes [26], podiatric surgery [27] and rheumatology [28].

### ‘Condition vs Complaint’

Whilst the podiatrists interviewed in this study believe that their services are valued and appreciated by their patients, they express concern that only certain people can access NHS podiatry care in the UK. Interestingly, despite the evidencing of outcomes, cost effectiveness and quality of services being currently so important in the procurement and commissioning of NHS services, there continues to be a paucity of published evidence to show the ‘value’ that patients attribute to UK podiatry services and the interventions that podiatrists use, both in respect to its significant importance amongst healthcare delivery and its impact upon patients’ quality of life. Patients who can afford it may choose to seek foot care expertise from the private sector, yet this excludes many. The implications are made clear. In the UK, the majority of NHS podiatry care is initiated from within the primary care sector through referral from GPs [7]. Podiatric clinicians in this investigation suggested that Clinical Commissioning Groups (CCGs), comprising primary care GPs, were much less well informed about the potential health gains to be made through referral to podiatry than perhaps other services, or in diabetes care, where MDT working enhances professional profiles. As a result, podiatrists in this investigation noted that community referral to podiatry is less well targeted than it might be, given the lack of understanding of the roles and skills of modern podiatrists [29] among GPs and other commissioners in primary care. This is reflective of recent analysis of GP referral patterns for foot pain, which found that the majority went to orthopaedics [30]. Interestingly this reflects the cultural and socio-historical context of the allied health professions, which occupied a lesser position within a complex health division of labour throughout most of the twentieth century [23, 31].

A sense of frustration was consistent through the discussions on the disparity across the UK of accessibility to NHS podiatry services and the inequity between areas, patient groups and commissioning bodies. Eligibility criteria, and the use of ‘tick box’ access, in combination with a national public health drive, appears to have resulted in open eligibility to podiatry services for some patients, but limited or barred access for other patient groups who may have an equal or greater podiatric need.

Whilst this is positive and inclusive for ‘eligible’ people, it excludes others. This allows access to podiatry services for people who may not need specialist podiatric intervention at the time, yet are able to access care because they have a specific condition, albeit not affecting their feet. This, in turn appears to be causing inequities of condition over complaint. Barriers towards accessing podiatry care for individuals who have arthritis have been previously highlighted in other countries [32–34]. This is consistent with findings that the provision of foot care for people with rheumatoid arthritis is not driven by foot health characteristics such as foot pain or foot related disability [32, 35] and indicates that the role of podiatry in the prevention of deterioration of foot health is partially, but not fully, recognised.

Alone, medical or podiatric pathology can place a person at mild, moderate or severe podiatric risk status. With comorbidities, this risk increases, in some cases significantly [36, 37]. Podiatrists are aware of the risk status and vulnerability of patients who do not have their podiatric needs met, including those who are ineligible for NHS care but cannot afford private sector care. From the concerns podiatrists expressed on behalf of their service users, came some consideration of alternative foot care options that the public access. Private practice was represented positively in providing valuable services, from both private sector and NHS practitioners, including suggestion that it enables NHS services to ‘cope’. Alternative foot health providers were broached, including charitable organisations and ‘nail bars’, where concerns were aired from both NHS and private sector practitioners, as were the incongruities of jointly managing patients between multiple providers. This echoes the views of other experts advocating podiatric intervention for those with foot health vulnerability and pathology due to long term health conditions [36–39]. This, in turn, synchronises with NHS England’s (2014) 5 Year Forward View [40] for a healthcare system that demonstrates improvements in service outcomes, improves preventative care, enables the frail and elderly to stay healthy, independent and access individualised, person centred care.

Frustration was reported over the prioritisation of UK NHS service provision according to the contract type, with commissioned services having priority over service level agreements, often prioritising one patient group, or clinic, over another. Furthermore confusion was expressed, by podiatrists in our investigations, on behalf of service users not eligible to access podiatry services when acquaintances were. Feedback from people who have chronic conditions is consistent with this, indicating that patients are not accessing foot healthcare [1–3, 41] and that they are confused over referral pathways to podiatry services [2, 42, 43].

### Transforming & sustaining podiatry

Since the inception of podiatry in the early part of the twentieth century, podiatry models of care within the UK NHS have remained fairly static and the model of access and care within private practice has remained stable within the realms of financial accessibility [7]. Despite that, demand has shifted towards acute levels of care within the UK NHS. Changes to service access in the 1990s has led to resource allocation, guidance and prioritisation for patients with complex acute care needs, predominantly diabetes [19].

Provision of podiatry services for individuals with arthritis is an area that lacks guidance. Whilst participants of this study acknowledged the presence of some foot health guidelines for people with arthritic conditions, referring specifically to the Arthritis and Musculoskeletal Alliance (ARMA) and NICE guidelines [44] for people with arthritis, they reported little recognition of, nor sufficient emphasis placed on, the use of these relevant guidelines by non-podiatry health professions or patients. The deficiency in specific guidance for foot health for people arthritis conditions appears to be in contrast to the vast plethora of guidance available for foot care services and intervention for people with diabetes [19]. Indeed, current literature suggests existing foot health guidelines for people with arthritic conditions are not being utilised to their fullest advantage and there is a recognised need to improve the implementation of such guidance [45]. Whilst new models of foot care for individuals who have inflammatory arthritis have been proposed [43, 46, 47] the recognition, development and implementation of guidance and pathways for other long term conditions that affect the feet (including osteoarthritis) is necessitated in a similar format to those for people with diabetes.

Podiatrists in this investigation considered that current UK health recommendations on recognition, transformation, sustainability and public health [40, 48] to be a timely opportunity for the profession to promote and re-establish podiatry’s full scope of practice to all stakeholders (patients, service managers, commissioners and other health professionals). They too encourage the development of new care models for podiatry, to meet the burden of foot pain in the UK and demands of modern healthcare. Podiatry has long been encouraged to become more versatile and involved in preventative healthcare [7, 49] and this now coordinates with recommendations for all AHPs [48]. Podiatrists interviewed believed change was needed, to sustain the provision of services and profession.

### Strengths and potential limitations

This study examined the perceptions of podiatrists in two regions of the UK, as a potentially representative snapshot. By using the experiences of 2 diverse groups of podiatrists from two disparate regions, rich text and themes have been generated. Limitations are acknowledged as both are in England, therefore data may not be wholly representative of the four home UK nations (England, Scotland, Wales and Northern Ireland) meaning proposed themes may be more or less significant in other areas. This may, however, align with high degrees of variation in specialist rheumatology service provision across the UK, wherein podiatry remains a notably poorly represented profession [50].

## Conclusion

The burden of foot pain for individuals living with arthritis in the UK is not insubstantial, yet many cannot access NHS podiatry services [7, 51]. The key themes derived from the podiatry clinician focus groups suggest that there are a variety of factors influencing demand for, and burden of, foot pain within the UK. Primarily, participants expressed frustration on having a service that accepts and treats patients according to their condition, rather than their complaint. Secondly, concern was conveyed over variations in stakeholders’ understanding of what podiatry is and what podiatrists aim to achieve for patients. Essentially, podiatrists interviewed called for the reform of the current accessibility to services to one that matches the foot care needs of individual patients. Clinicians are keen to explore alternative ways to promote podiatry services for procurement and new models of service provision, to be more reflective of people’s individual circumstances and believe that setting and implementing nationally set guidance is the optimal way to inform commissioning of podiatry services towards a more inclusive service provision.

## Abbreviations

ARMA: Arthritis and Musculoskeletal Alliance
GP: General Practitioner
NICE: National Institute for Health and Care Excellence
